# Residual Disparities in US Adults Hospitalized due to All-Cause Pneumonia Following Implementation of Childhood 13-Valent Pneumococcal Conjugate Vaccine in National Immunization Program

**DOI:** 10.1093/ofid/ofag528

**Published:** 2026-08-24

**Authors:** Mark H Rozenbaum, Ahuva Averin, Derek Weycker, Rotem Lapidot, Amanda Miles, Maria J Tort, Paula Peyrani, Jeffrey Vietri, Alexander Lonshteyn, Stephen I Pelton

**Affiliations:** HTA Value & Evidence, Pfizer Inc, Capelle A/d Ijssel, The Netherlands; Evidence & Strategy, Avalere Health, Washington, District of Columbia, USA; Evidence & Strategy, Avalere Health, Washington, District of Columbia, USA; Division of Infectious Diseases, Rambam Health Care Campus, Haifa, Israel; Faculty of Medicine, Technion, Israel Institute of Technology, Haifa, Israel; Department of Pediatrics, Boston Medical Centre, Boston, Massachusetts, United States; Department of Pediatrics, Boston University Chobanian & Avedisian School of Medicine, Boston, Massachusetts, United States; Real World Evidence and Epidemiology, Pfizer Inc, New York, New York, United States; US Vaccines Medical Affairs, Pfizer Inc, Collegeville, Pennsylvania, United States; US Vaccines Medical Affairs, Pfizer Inc, Collegeville, Pennsylvania, United States; HTA Value & Evidence, Pfizer Inc, Collegeville, Pennsylvania, United States; Evidence & Strategy, Avalere Health, Washington, District of Columbia, USA; Department of Pediatrics, Boston Medical Centre, Boston, Massachusetts, United States; Department of Pediatrics, Boston University Chobanian & Avedisian School of Medicine, Boston, Massachusetts, United States

**Keywords:** adults, comorbidity, herd immunity, pneumococcal vaccines, socioeconomic factors

## Abstract

**Background:**

Use of 13-valent pneumococcal conjugate vaccine (PCV13) has reduced the burden of pneumococcal disease among both children and adults. Research has indicated that some population subgroups have benefited more than others. This study estimated rates of inpatient all-cause pneumonia among US adults by age, race, comorbidity profile, and household income (HHI), before and after universal recommendation for PCV13 in children and targeted recommendation for use in adults.

**Methods:**

A retrospective observational study was conducted using Optum's de-identified Clinformatics Data Mart Database (2007–2019). Adults aged ≥18 years were stratified by age, race, comorbidity profile (low-risk, at-risk/high-risk), and annual household income. Inpatient all-cause pneumonia episodes were identified via relevant *International Classification of Diseases* (Ninth and Tenth Revisions) codes. Rates per 100 000 person-years and incidence rate ratios (IRRs) were calculated for the pre-PCV13 (2008–2009), peri-PCV13 (2010–2012), post-PCV13#1 (2013–2016), and post-PCV13#2 (2017–2019) periods.

**Results:**

Among 38.7 million US adults, inpatient all-cause pneumonia rates decreased from the pre-PCV13 to post-PCV13#2 period among all age groups (eg, 72.9 to 38.2 among 18- to 49-year-olds and 1621.8 to 1326.0 among ≥75-year-olds). Irrespective of age, rates generally decreased from the pre-PCV13 to post-PCV13#2 period within subgroups defined by race, comorbidity profile, and HHI; the greatest relative reductions were observed among persons aged 18–49 years. During the post-PCV13#2 period, the highest rates of inpatient all-cause pneumonia persisted in Black adults, at-risk/high-risk adults, and adults with HHI <$100 000.

**Conclusions:**

Despite reductions in inpatient all-cause pneumonia following pediatric PCV13 introduction, residual disparities persist for Black, at-risk/high-risk, and low- and middle-income adults. Strategies to reduce persistent disparities in disease burden among affected adults should be considered and implemented.

## Teaser

Introduction of the 13-valent pneumococcal conjugate vaccine reduced inpatient all-cause pneumonia rates among US adults, but significant disparities persist for Black, high-risk, and low-/middle-income groups. Targeted strategies are needed to address these ongoing inequities in disease burden.

Pneumococcal disease is an acute infection caused by *Streptococcus pneumoniae*, resulting in significant disease-related morbidity and mortality. Among adults, those who are older and those with certain chronic medical conditions are particularly susceptible and have the highest observed disease burden [1–3]. Prior research has also indicated that racial or socioeconomic factors impact pneumococcal disease risk among adults in the United States [4–6].

The 7-valent pneumococcal conjugate vaccine (PCV7) was recommended for routine use among all infants by the US Centers for Disease Control and Prevention’s Advisory Committee on Immunization Practices (ACIP) in 2000 [7]. Following PCV7 introduction, substantial declines in invasive pneumococcal disease (IPD) and pneumonia (PNA) hospitalizations were observed among both children and adults, largely driven by reductions in vaccine-type disease due to both direct and indirect (herd) effects of infant vaccination [8–10]. In 2010, the ACIP recommended the 13-valent pneumococcal conjugate vaccine (PCV13) for prevention of pneumococcal disease among infants and children in the United States [11]. In 2012, the ACIP recommendation was expanded to include adults aged ≥18 years with immunocompromising conditions, and in 2014, the recommendation was further expanded to include all adults aged ≥65 years [12, 13]. In 2024, ACIP expanded the routine recommendation for pneumococcal conjugate vaccine (PCV) to all PCV-naïve adults aged ≥50 years, and both pediatric and adult recommendations have been updated to include use of higher-valent PCVs [14, 15].

The rollout of the pediatric PCV13 immunization program significantly influenced pneumococcal disease prevention and led to studies assessing its impact on adults. Although previous studies have investigated socioeconomic, racial, and sex disparities among adults with regard to pneumococcal disease rates after the introduction of PCV13 [5, 16], these studies focused primarily on IPD and did not address nonbacteremic PNA, which is the most prevalent pneumococcal syndrome among adults. The current study evaluated trends in adult rates of inpatient all-cause PNA from 2008 to 2019, spanning the period before and after introduction of pediatric PCV13 (2010). We additionally examined whether disparities in inpatient all-cause PNA rates—by demographic and clinical characteristics as well as household income—persisted later in the study period.

## METHODS

### Study Design and Data Source

A retrospective observational cohort design was employed using data spanning January 2007 through December 2019 from Optum's de-identified Clinformatics Data Mart Database (CDM). Optum's de-identified CDM is a national database comprising insurer-provided medical (ie, facility and professional service) and outpatient pharmacy claims data from >15 million covered lives annually in all 50 US states. Insurers contributing healthcare claims to the CDM administer both commercial and Medicare Advantage health plans, which may be employer sponsored or purchased by individuals. Medical claims include diagnoses, procedures, dates and places of service, and provider category. Pharmacy claims include the prescription product, date of prescription, days supplied, and quantity supplied. All administrative claims are verified, adjudicated, adjusted, and de-identified by Optum prior to inclusion in the database. Demographic and eligibility information—including age, sex, race, and household income (HHI)—are also available in the CDM.

### Study Population

The study population comprised of US adults aged ≥18 years with at least 1 year of continuous healthcare coverage during the period spanning 1 January 2007 through 31 December 2019. The day after the end date of the initial 1-year continuous enrollment period was designated as the “index date.” In the case of persons with multiple unique qualifying periods of healthcare coverage, each period was considered independently in analyses.

### Study Variables

Baseline characteristics were ascertained at index and included age, sex, race (Asian, Black, Hispanic, White, unknown), comorbidity profile, and HHI. Comorbidity profile was defined based on the presence or absence of conditions that increase the risk of pneumococcal disease, using the same approach as described in a previous study [17]. “High-risk” comprised immunocompromised persons based on the presence of ≥1 immunosuppressive condition, “at-risk” comprised immunocompetent persons with ≥1 chronic medical condition, and “low-risk” comprised immunocompetent persons without chronic medical conditions. HHI categories were defined as follows: <$50 000 (low), $50 000–$99 999 (middle), ≥$100 000 (high), and unknown/missing. Cut points of $50 000 and $100 000 were selected from among the income categories available in the source data to yield approximately equal-sized groups within the study population. Due to limitations of healthcare claims data, adult vaccination status could not be reliably ascertained from claims data due to high rates of plan turnover (disenrollment) in commercial and Medicare Advantage health plans, which limits the ability to construct complete longitudinal vaccination histories.

Episodes of inpatient all-cause PNA were identified based on hospitalizations with corresponding diagnosis codes; cases with evidence of invasive disease were excluded (Supplementary Appendix). Episodes were ascertained on a monthly basis, beginning on the index date and ending on the last date of health plan enrollment or last date of the study period, whichever occurred first. All qualifying hospitalizations occurring within 30 days of each other were considered part of the same episode.

### Statistical Analysis

Baseline characteristics of the study population were summarized via counts and percentages. Rates of inpatient all-cause PNA were estimated during the pre-PCV13 period (2008–2009), peri-PCV13 period (2010–2012), post-PCV13#1 period (2013–2016), and post-PCV13#2 period (2017–2019), respectively, by age group as well as within age-specific subgroups defined on race, comorbidity profile, and HHI. Three segments of follow-up were employed to evaluate temporal trends in rates of adult all-cause PNA across the 10-year period following the approval of PCV13.

Rates were reported per 100 000 person-years and were adjusted for differential follow-up using a population-based approach (ie, total number of episodes divided by total number of person-days of observation, multiplied by 365.25); techniques of nonparametric bootstrapping were used to estimate 95% confidence intervals (CIs). Poisson regression models were employed to estimate differences in disease incidence (ie, incidence rate ratios [IRRs]) between periods and subgroups of interest, respectively.

## RESULTS

### Baseline Characteristics

Among the 38.7 million adults aged ≥18 years in the study database who met the study criteria, 55% were aged 18–49 years, 23% were aged 50–64 years, 14% were aged 65–74 years, and 8% were aged ≥75 years; 51% were female (Table 1). The population was 66% White, 11% Hispanic, 10% Black, and 5% Asian; race was missing for 6%–10% of adults. While the majority of the overall population was low-risk (79%), the percentage of individuals with comorbidities increased with increasing age, with 53% of ≥75-year-olds categorized as at-risk/high-risk. Study population was evenly distributed across HHI thresholds, with 25%, 28%, and 28% having respective annual HHI of <$50 000, $50 000–$99 999, and ≥$100 000; HHI was missing for 11%–23% of adults.

**Table 1.** Baseline Characteristics of the Study Population

Characteristic	All (n = 38 709 763)	Age 18–49 y (n = 21 315 911)	Age 50–64 y (n = 8 758 170)	Age 65–74 y (n = 5 503 950)	Age ≥75 y (n = 3 131 732)
Sex					
Female	51.4%	49.7%	50.5%	54.8%	59.2%
Male	48.6%	50.2%	49.4%	45.2%	40.8%
Race					
Asian	4.9%	6.2%	3.2%	3.8%	3.0%
Black	9.9%	9.8%	9.7%	10.4%	10.3%
Hispanic	10.7%	12.0%	8.5%	9.8%	9.0%
White	65.8%	62.1%	70.7%	69.1%	71.3%
Unknown/missing	8.7%	9.9%	7.9%	6.9%	6.4%
Insurance type					
Commercial	77.4%	98.8%	89.7%	15.4%	6.9%
Medicare Advantage	22.5%	1.1%	10.2%	84.5%	93.1%
Unknown/missing	0.1%	0.1%	0.1%	0.0%	0.0%
Comorbidity profile					
Low-risk	78.7%	90.9%	73.5%	57.9%	47.2%
At-risk	17.6%	7.9%	22.0%	34.3%	41.6%
Alcoholism	0.3%	0.3%	0.5%	0.4%	0.2%
Asthma	2.7%	2.1%	3.1%	3.7%	3.5%
Diabetes	8.5%	2.5%	11.5%	19.8%	20.9%
Heart disease	5.9%	0.9%	6.6%	13.7%	24.3%
Liver disease	0.8%	0.4%	1.2%	1.5%	0.9%
Lung disease	2.6%	0.4%	3.1%	6.5%	9.7%
Smoker	3.2%	2.6%	4.4%	3.9%	1.9%
High-risk	3.7%	1.2%	4.6%	7.9%	11.2%
Asplenia	0.0%	0.0%	0.0%	0.0%	0.0%
Blood cell disorders	0.1%	0.1%	0.1%	0.2%	0.3%
Cerebrospinal fluid leak	0.0%	0.0%	0.0%	0.0%	0.0%
Cochlear implant	0.0%	0.0%	0.0%	0.0%	0.0%
Congenital immunodeficiency	0.2%	0.1%	0.3%	0.4%	0.3%
Human immunodeficiency virus	0.2%	0.2%	0.3%	0.1%	0.0%
Malignancy	2.9%	0.7%	3.5%	6.5%	9.0%
Renal disease	0.4%	0.1%	0.4%	0.9%	2.0%
Nephrotic syndrome	0.0%	0.0%	0.0%	0.1%	0.1%
Organ transplant	0.1%	0.1%	0.2%	0.2%	0.1%
At-risk/high-risk	21.3%	9.1%	26.5%	42.1%	52.8%
Household income (annual)					
<$50 000	25.0%	20.5%	22.7%	35.9%	42.6%
$50 000–$99 999	27.9%	25.2%	30.1%	34.4%	28.5%
≥$100 000	28.0%	31.6%	30.9%	18.5%	12.4%
Unknown/missing	19.1%	22.7%	16.4%	11.2%	16.4%

### Rates and IRRs of Inpatient All-Cause PNA by Age

Rates of inpatient all-cause PNA were lower during the post-PCV13 periods compared to the pre-PCV13 period across all age groups, with the greatest relative reduction in adults aged 18–49 years, followed by those aged 65–74, ≥75, and 50–64 years (Table 2). For adults aged 18–49 years, rates of hospitalized all-cause PNA per 100 000 person-years (95% CI) decreased from 72.9 (71.1–74.8) in the pre-PCV13 period to 38.2 (37.1–39.3) in the post-PCV13#2 period; rates decreased from 250.4 (245.5–255.3) to 225.2 (221.6–228.8) for adults aged 50–64 years, from 615.5 (603.2–628.1) to 490.4 (485.0–495.9) for adults aged 65–74 years, and from 1621.8 (1600.7–1643.2) to 1326.0 (1316.8–1335.3) for adults aged ≥75 years. Adults 18–49 years of age had the largest relative decrease in inpatient all-cause PNA rates from the pre-PCV13 period to the post-PCV13#2 period, with an IRR of 0.52 (95% CI, .50–.54), equal to a relative reduction of 48%. Among the older age groups, the IRRs for the post-PCV13#2 period (vs pre-PCV13 period) ranged from 0.80 (95% CI, .78–.82) to 0.90 (95% CI, .88–.92), corresponding to relative reductions of 20% and 10%, respectively.

**Table 2.** Rates of Inpatient All-Cause Pneumonia and Disease Incidence Rate Ratios by Age

Age Group	Pre-PCV13 (2008–2009)	Peri-PCV13 (2010–2012)	Post-PCV13#1 (2013–2016)	Post-PCV13#2 (2017–2019)
18–49 y				
Rate/100 000 PY (95% CI)	72.9 (71.1–74.8)	62.7 (61.2–64.1)	49.3 (48.2–50.5)	38.2 (37.1–39.3)
IRR (95% CI)	referent	0.86 (.83–.89)	0.68 (.65–.70)	0.52 (.50–.54)
No. of PY	8 259 629	11 327 739	14 437 616	12 030 046
50–64 y				
Rate/100 000 PY (95% CI)	250.4 (245.5–255.3)	258.5 (254.4–262.6)	240.9 (237.5–244.4)	225.2 (221.6–228.8)
IRR (95% CI)	referent	1.03 (1.01–1.06)	0.96 (.94–.99)	0.90 (.88–.92)
No. of PY	4 057 781	5 999 597	7 838 216	6 635 761
65–74 y				
Rate/100 000 PY (95% CI)	615.5 (603.2–628.1)	652.9 (643.5–662.3)	561.4 (555.1–567.9)	490.4 (485.0–495.9)
IRR (95% CI)	referent	1.06 (1.03–1.09)	0.91 (.89–.93)	0.80 (.78–.82)
No. of PY	1 526 772	2 846 387	5 302 763	6 303 718
≥75 y				
Rate/100 000 PY (95% CI)	1621.8 (1600.7–1643.2)	1636.7 (1621.3–1652.4)	1437.3 (1426.6–1448.2)	1326.0 (1316.8–1335.3)
IRR (95% CI)	referent	1.01 (.99–1.03)	0.89 (.87–.90)	0.82 (.81–.83)
No. of PY	1 378 740	2 600 546	4 741 103	5 961 414

Abbreviations: CI, confidence interval; IRR, incidence rate ratio; PCV13, 13-valent pneumococcal conjugate vaccine; PY, person-years.

### Age-Specific Rates and IRRs of Inpatient All-Cause PNA Comparing Pre-PCV13 Versus Post-PCV13#2 Periods by Race, Comorbidity Profile, and HHI

Across all age groups and time periods, rates of inpatient all-cause PNA were highest among Black, at-risk/high-risk, and low HHI groups (Table 3; Supplementary Tables 1–4). Among those aged 18–49 years, inpatient all-cause PNA rates for all subgroups decreased over time. IRRs for the post-PCV13#2 period (compared to pre-PCV13) were similar across all subgroups defined by race, comorbidity profile, and HHI in this age group, ranging from 0.46 (95% CI, .44–.49) to 0.66 (95% CI, .62–.69). Among adults ≥50 years of age, rates decreased from the pre-PCV13 to post-PCV13#2 period in two-thirds of the subgroups defined by race, comorbidity profile, and HHI, while remaining stable or increasing in others. For example, among adults aged 50–64 years, rates increased for the low-income group and showed no significant change for the Asian, Black, and Hispanic groups. In addition, among adults aged ≥75 years, the rates increased for the middle-income group and remained similar for the Hispanic, low-risk, and high-income groups from the pre-PCV13 to post-PCV13#2 period.

**Table 3.** Age-Specific Rates of Inpatient All-Cause Pneumonia and Disease Incidence Rate Ratios by Race, Comorbidity Profile, and Household Income

Characteristic	Age 18–49 y		Age 50–64 y		Age 65–74 y		Age ≥75 y	
	Pre-PCV13	Post-PCV13#2	Pre-PCV13	Post-PCV13#2	Pre-PCV13	Post-PCV13#2	Pre-PCV13	Post-PCV13#2
Race								
Asian								
Rate/100 000 PY (95% CI)	29.8 (25.1–35.4)	14.6 (12.2–17.3)	97.4 (81.9–115.8)	89.6 (78.7–102.0)	328.9 (280.4–385.8)	241.4 (223.1–261.2)	992.1 (895.2–1099.6)	761.5 (724.9–799.9)
IRR (95% CI)	referent	0.49 (.38–.62)	referent	0.92 (.74–1.14)	referent	0.73 (.61–.88)	referent	0.77 (.68–.86)
No. of PY	435 679	879 645	131 392	254 480	45 908	255 616	36 587	208 542
Black								
Rate/100 000 PY (95% CI)	115.3 (107.9–123.3)	72.0 (67.3–76.9)	367.3 (348.2–387.6)	389.1 (374.6–404.2)	877.9 (830.5–927.9)	672.3 (653.2–692.0)	1998.7 (1918.9–2081.7)	1399.6 (1369.2–1430.6)
IRR (95% CI)	referent	0.62 (.57–.69)	referent	1.06 (.99–1.13)	referent	0.77 (.72–.82)	referent	0.70 (.67–.73)
No. of PY	753 542	1 190 913	363 703	686 667	142 387	685 667	115 877	569 741
Hispanic								
Rate/100 000 PY (95% CI)	61.0 (56.1–66.4)	29.4 (26.9–32.1)	193.1 (177.8–209.7)	177.0 (167.4–187.1)	414.1 (382.2–448.7)	383.4 (368.7–398.6)	1097.4 (1041.4–1156.4)	1046.0 (1020.8–1071.9)
IRR (95% CI)	referent	0.48 (.43–.54)	referent	0.92 (.83–1.01)	referent	0.93 (.85–1.01)	referent	0.95 (.90–1.01)
No. of PY	878 042	1 695 331	291 555	696 141	144 404	660 968	127 670	613 843
White								
Rate/100 000 PY (95% CI)	72.5 (70.2–74.9)	37.8 (36.5–39.2)	244.1 (238.5–249.9)	213.8 (209.7–218.0)	618.5 (604.0–633.4)	487.4 (480.9–494.0)	1673.5 (1648.5–1698.8)	1377.2 (1366.2–1388.3)
IRR (95% CI)	referent	0.52 (.50–.55)	referent	0.88 (.85–.90)	referent	0.79 (.77–.81)	referent	0.82 (.81–.84)
No. of PY	5 186 342	7 692 390	2 882 269	4 764 665	1 098 053	4 391 035	1 012 935	4 308 101
Comorbidity profile								
Low-risk								
Rate/100 000 PY (95% CI)	45.4 (43.9–47.0)	21.1 (20.2–21.9)	118.0 (114.3–121.9)	86.3 (83.7–88.9)	229.1 (220.0–238.5)	209.4 (204.8–214.1)	817.2 (798.5–836.3)	804.8 (795.2–814.6)
IRR (95% CI)	referent	0.46 (.44–.49)	referent	0.73 (.70–.76)	referent	0.91 (.87–.96)	referent	0.98 (.96–1.01)
No. of PY	7 544 934	11 085 251	3 130 218	5 039 194	1 022 814	3 713 791	876 927	3 269 940
At-risk/high-risk								
Rate/100 000 PY (95% CI)	363.5 (349.8–377.8)	238.8 (229.1–248.8)	697.1 (680.3–714.3)	663.7 (651.2–676.4)	1400.1 (1367.8–1433.2)	893.4 (881.9–905.0)	3027.8 (2980.1–3076.3)	1959.2 (1942.5–1976.0)
IRR (95% CI)	referent	0.66 (.62–.69)	referent	0.95 (.92–.98)	referent	0.64 (.62–.66)	referent	0.65 (.64–.66)
No. of PY	714 695	944 796	927 563	1 596 567	503 957	2 589 927	501 813	2 691 474
Household income								
<$50 000								
Rate/100 000 PY (95% CI)	111.3 (105.7–117.2)	70.0 (66.9–73.2)	378.8 (365.1–393.1)	442.5 (432.5–452.7)	701.5 (680.1–723.5)	662.3 (651.6–673.1)	1522.0 (1491.2–1553.5)	1412.1 (1397.6–1426.7)
IRR (95% CI)	referent	0.63 (.59–.67)	referent	1.17 (1.12–1.22)	referent	0.94 (.91–.98)	referent	0.93 (.91–.95)
No. of PY	1 305 703	2 713 958	740 680	1 669 563	570 659	2 219 362	602 154	2 559 726
$50 000–$99 999								
Rate/100 000 PY (95% CI)	72.7 (69.1–76.5)	35.6 (33.6–37.7)	227.0 (218.8–235.5)	187.5 (181.7–193.5)	430.4 (412.4–449.1)	392.1 (384.2–400.2)	1073.4 (1039.1–1108.8)	1198.3 (1183.6–1213.3)
IRR (95% CI)	referent	0.49 (.45–.53)	referent	0.83 (.79–.87)	referent	0.91 (.87–.96)	referent	1.12 (1.08–1.16)
No. of PY	2 055 473	3 206 952	1 256 074	2 076 268	492 374	2 358 146	340 323	2 087 810
≥$100 000								
Rate/100 000 PY (95% CI)	47.9 (45.4–50.5)	24.0 (22.6–25.4)	133.8 (127.6–140.2)	86.1 (82.5–89.9)	341.4 (319.7–364.5)	275.2 (266.5–284.2)	1020.1 (971.9–1070.6)	1073.2 (1052.5–1094.4)
IRR (95% CI)	referent	0.50 (.46–.54)	referent	0.64 (.60–.69)	referent	0.81 (.75–.87)	referent	1.05 (1.00–1.11)
No. of PY	2 902 456	4 525 189	1 309 027	2 450 404	260 718	1 338 609	161 066	935 202

Abbreviations: CI, confidence interval; IRR, incidence rate ratio; PCV13, 13-valent pneumococcal conjugate vaccine; PY, person-years.

### Residual Disparities During the Post-PCV13#2 Period

Although reductions in risk of inpatient all-cause PNA were observed from the pre-PCV13 to post-PCV13#2 period within most population groups, we identified specific groups with a disproportional burden of disease during the post-PCV13#2 period (Table 4). Notably, Black individuals in the 18- to 49-year-old age group had nearly double the rate of disease compared to White individuals (IRR vs White: 1.90 [95% CI, 1.76–2.05]); this disparity between Black and White persons decreased monotonically with increasing age and persisted among adults aged 50–74 years, but was not significant among those aged ≥75 years (IRR, 1.02 [95% CI, .99–1.04]). Other race-defined groups had IRRs indicating lower rates of disease compared to White persons. Risk of inpatient all-cause PNA also remained markedly higher in the post-PCV13#2 period for at-risk/high-risk adults compared to low-risk adults, though IRR decreased with increasing age, from 11.33 (95% CI, 10.69–12.01) for those aged 18–49 years to 2.43 (95% CI, 2.40–2.47) for those aged ≥75 years. Lower HHI was also a persistent risk factor, with IRRs for low-income and middle-income (vs high-income) households ranging from 1.32 (95% CI, 1.29–1.35) to 5.14 (95% CI, 4.90–5.39) and from 1.12 (95% CI, 1.09–1.14) to 2.18 (95% CI, 2.07–2.30), respectively. Disparities related to HHI were most pronounced among adults aged 50–64 years.

**Table 4.** Age-Specific Incidence Rate Ratios of Inpatient All-Cause Pneumonia in the Post-PCV13#2 Period by Race, Comorbidity Profile, and Household Income

Characteristic	Age 18–49 y	Age 50–64 y	Age 65–74 y	Age ≥75 y
Race				
Asian	0.38 (.32–.46)	0.42 (.37–.48)	0.50 (.46–.54)	0.55 (.53–.58)
Black	1.90 (1.76–2.05)	1.82 (1.74–1.90)	1.38 (1.34–1.42)	1.02 (.99–1.04)
Hispanic	0.78 (.71–.85)	0.83 (.78–.88)	0.79 (.75–.82)	0.76 (.74–.78)
White	referent	referent	referent	referent
Unknown	0.92 (.79–1.06)	1.25 (1.15–1.36)	1.16 (1.10–1.21)	1.04 (1.00–1.07)
Comorbidity profile				
Low-risk	referent	referent	referent	referent
At-risk/high-risk	11.33 (10.69–12.01)	7.69 (7.43–7.97)	4.27 (4.16–4.38)	2.43 (2.40–2.47)
Household income				
<$50 000	2.92 (2.71–3.14)	5.14 (4.90–5.39)	2.41 (2.32–2.49)	1.32 (1.29–1.35)
$50 000–$99 999	1.48 (1.37–1.61)	2.18 (2.07–2.30)	1.42 (1.37–1.48)	1.12 (1.09–1.14)
≥$100 000	referent	referent	referent	referent
Unknown/missing	1.23 (1.10–1.37)	4.10 (3.84–4.38)	3.08 (2.94–3.23)	1.93 (1.87–1.99)

Data are shown as incidence rate ratio (95% confidence interval).

Abbreviation: PCV13, 13-valent pneumococcal conjugate vaccine.

## DISCUSSION

The present retrospective study utilized healthcare claims from >38 million adults to estimate rates of inpatient all-cause PNA among groups defined by age as well as within age-specific groups defined by race, comorbidity profile, and HHI, before and after ACIP recommendations for pediatric use of PCV13. Study findings indicate that rates of disease were lower during the post-PCV13#2 period (2017–2019) compared to the pre-PCV13 period for all age groups, though exceptions were observed when considering select groups of older adults (eg, low-income adults aged 50–64 years and middle-income adults aged ≥75 years). Adults aged 18–49 years had the largest relative decrease (48%) in inpatient all-cause PNA following the introduction of pediatric PCV13, while more modest reductions of 10%–20% were observed across older age groups. Despite reductions in disease over time, Black, at-risk/high-risk, and low- and middle-income adults bore a disproportionate burden of disease compared to their counterparts in other race-, risk-, and income-based groups during the post-PCV13#2 period.

Findings from the present study regarding age and comorbidity partially align with previous US studies of PCV13's indirect effect and direct protection against adult inpatient all-cause PNA. Pelton et al (2019) analyzed US health insurance claims and reported that rates of all-cause PNA hospitalizations between the pre-PCV13 (2007–2010) and post-PCV13 (2013–2015) periods generally declined among adults <75 years of age and across risk groups, but increased among those aged ≥75 years [18]. Similar to our study, Pelton et al observed that the greatest decrease (19%) in disease burden was among healthy persons aged 18–49 years and that the relative reduction in hospitalization rates from pre- to post-PCV13 was smaller among older adults and those with comorbidities compared to younger, healthier adults. We hypothesize that these findings reflect waning immunity and a decline in immune system function (ie, immunosenescence).

In a study of the earlier post-PCV13 time period, Simonsen et al (2014) analyzed private hospital discharge data and concluded that all-cause PNA hospitalization rates in the United States declined by 12% among adults aged 18–39 years within the first 2 years after PCV13 introduction (2011–2012), but no significant reduction was observed among adults aged 40–64 and ≥65 years [19]. In addition, a US claims study by Tong et al (2018) observed a significant reduction in overall rates of PNA (inpatient, outpatient, and emergency department) between 2008–2009 and 2014 across all age groups, except for adults aged ≥85 years, among whom rates increased [20]. The results for younger adults from Simonsen et al and Tong et al align with our findings of a 14% and 32% decrease in inpatient all-cause PNA among adults aged 18–49 years during the peri-PCV13 (2010–2012) and post-PCV13#1 (2013–2016) periods, respectively, compared to the pre-PCV13 period (2008–2009).

Overall, findings from the cited research [18–20] indicate either no change or an increase in PNA rates among older age groups. This aligns with our results, which showed either a minor increase or no significant change in inpatient all-cause PNA among adults aged ≥50 years during the peri-PCV13 (2010–2012) versus pre-PCV13 (2008–2009) period (IRRs ranged from 1.01 [95% CI, .99–1.03] to 1.06 [95% CI, 1.03–1.09]). However, we observed a decline in inpatient all-cause PNA rates across all age groups during the post-PCV13#1 (2013–2016) and post-PCV13#2 (2017–2019) periods, extending beyond the time frames examined in the referenced studies. This reduction may reflect both enhanced herd effects due to increasing PCV13 coverage in successive birth cohorts and targeted immunization efforts among high-risk adults.

Evidence from other countries indicates that rates of all-cause PNA hospitalizations have decreased among older adults. A Canadian study (2018) using health insurance and hospital discharge data from 1992–2014 reported substantive relative reductions in both PNA hospitalizations and associated costs across most age groups, with the largest reductions among persons aged ≥65 years [21]. In England, Rodrigo et al (2015) conducted a prospective cohort study and observed a 15% relative reduction in adult community-acquired pneumonia (CAP) hospitalization post-PCV13 (2010–2013) versus pre-PCV13 (2008–2009) [22]. The largest absolute and relative reduction in CAP hospitalization occurred among adults aged ≥85 years.

In contrast, evidence on disparities in PNA between subgroups defined by racial, ethnic, and socioeconomic factors is sparse and currently limited to IPD. For example, Raman et al (2021) evaluated rates of IPD relative to pediatric PCV13 implementation among persons of all ages in Tennessee during the pre-PCV7 (1998–1999), pre-PCV13 (2001–2009), and post-PCV13 (2011–2016) periods [5]. Though differences in study design (eg, limited source population, distinct clinical manifestation of pneumococcal disease, and alternatively defined subgroups) inhibit a direct comparison of results, similar trends were observed, suggesting that the introduction of PCV13 was associated with substantial reductions in racial and socioeconomic disparities in IPD incidence. However, the authors concluded that burden of IPD remained higher among Black (vs White) people and the poorest households. A similar finding was noted in the present study for inpatient all-cause PNA among Black and lower HHI groups, with racial and income disparities (ie, IRRs) generally decreasing with increasing age, except for low- and middle-income adults aged 50–64 years.

We note several limitations of the current study. While the Optum CDM provides information on large numbers of patients across the United States, study results may not be generalizable to other populations or settings. People who are uninsured and those with other types of health insurance coverage (eg, Medicaid or fee-for-service Medicare) are not included in the study database, so our study population may not be representative of the total population of US adults in terms of demographic or socioeconomic characteristics. We also note that while similar operational algorithms have been implemented in prior research [2, 23–25], classification of patients based on their comorbidity profile and identifying episodes of PNA may have resulted in some misclassification bias. Race and HHI proxies in the Optum CDM are based on a mix of individual-level and ecological-level data and, to our knowledge, have not been evaluated against a gold standard. Further, it is likely that the reasons for declines in inpatient all-cause PNA are multifactorial, including the introduction of PCV13 as well as higher uptake of influenza vaccines, changes in practice patterns, expanded in-home medical services, reductions in smoking rates, and better chronic disease management. Relatedly, because our study outcome was inpatient all-cause PNA—not pneumococcal PNA—the attenuated reductions and small increases in PNA hospitalizations observed among certain subgroups of adults may reflect the growing proportion of all-cause PNA with nonpneumococcal etiologies or due to serotypes not covered by vaccines (ie, serotype replacement), which would not be expected to decline as a result of PCV13 vaccination. As well, our analysis period ended in 2019 and does not capture disease prevented through 2022–2023 when PCV13 was replaced by higher-valent PCVs. Finally, we note that while our study indirectly addresses real-world effectiveness of PCV13 by assessing reductions in episodes of all-cause hospitalized PNA, our study was not intended to explicitly estimate vaccine effectiveness and accordingly was not adjusted for potential confounders. Moreover, it should be noted that because uptake of adult vaccines relevant to PNA, such as pneumococcal and influenza vaccines, was not characterized in our study population, analyses stratified based on evidence of receipt (yes/no) of such vaccines were not conducted. As such, it is not possible to discern whether reductions in inpatient all-cause PNA among vaccine-eligible subgroups of adults were driven predominantly by direct effects of adult vaccination or indirect effects of pediatric vaccination or other factors.

## CONCLUSIONS

In the years following the 2010 and 2012 ACIP recommendations for the use of PCV13, rates of inpatient all-cause PNA in the United States declined compared to the years prior to widespread use. These declines were observed consistently across all age groups studied and, within age groups, across subgroups defined on patient characteristics, with 2 exceptions (aged 50–64 years with HHI <$50 000; aged ≥75 years with HHI $50 000–$99 999). Despite the reduction in cases, though, disparities in the burden of disease remain among certain population subgroups, including Black persons, those with at-risk/high-risk conditions, and persons with HHI <$100 000. Our findings highlight the importance of continually assessing and improving strategies to address these disparities.

## Supplementary Material

ofag528_Supplementary_Data

## Supplementary Data

Supplementary materials are available at *Open Forum Infectious Diseases* online. Consisting of data provided by the authors to benefit the reader, the posted materials are not copyedited and are the sole responsibility of the authors, so questions or comments should be addressed to the corresponding author.
