# Supplementary material for: Residual Disparities in US Adults Hospitalized due to All-Cause Pneumonia Following Implementation of Childhood 13-Valent Pneumococcal Conjugate Vaccine in National Immunization Program
Source: Open Forum Infect Dis. 2026 Aug 24;13(9):ofag528. doi: 10.1093/ofid/ofag528 (PMC13546781; doi:10.1093/ofid/ofag528)
Supplement: Supplementary file 1 [file ofag528_supplementary_data.docx]

**Supplemental Materials**

**Supplement -- Appendix.** Operational algorithms for identifying study outcomes*

**Supplement -- Table 1.** Rates of inpatient all-cause PNA and disease incidence rate ratios among adults aged 18-49 years by race, comorbidity profile, and household income

**Supplement -- Table 2.** Rates of inpatient all-cause PNA and disease incidence rate ratios among adults aged 50-64 years by race, comorbidity profile, and household income

**Supplement -- Table 3.** Rates of inpatient all-cause PNA and disease incidence rate ratios among adults aged 65-74 years by race, comorbidity profile, and household income

**Supplement -- Table 4.** Rates of inpatient all-cause PNA and disease incidence rate ratios among adults aged ≥75 years by race, comorbidity profile, and household income
